# Supplementary figures and images for: Smoothened turnover regulated by Hedgehog signaling in Drosophila
Source: bioRxiv. 2026 Jan 9:2026.01.08.698469. Preprint. [Version 1] doi: 10.64898/2026.01.08.698469 (PMC12803285; doi:10.64898/2026.01.08.698469)

Figure 1S

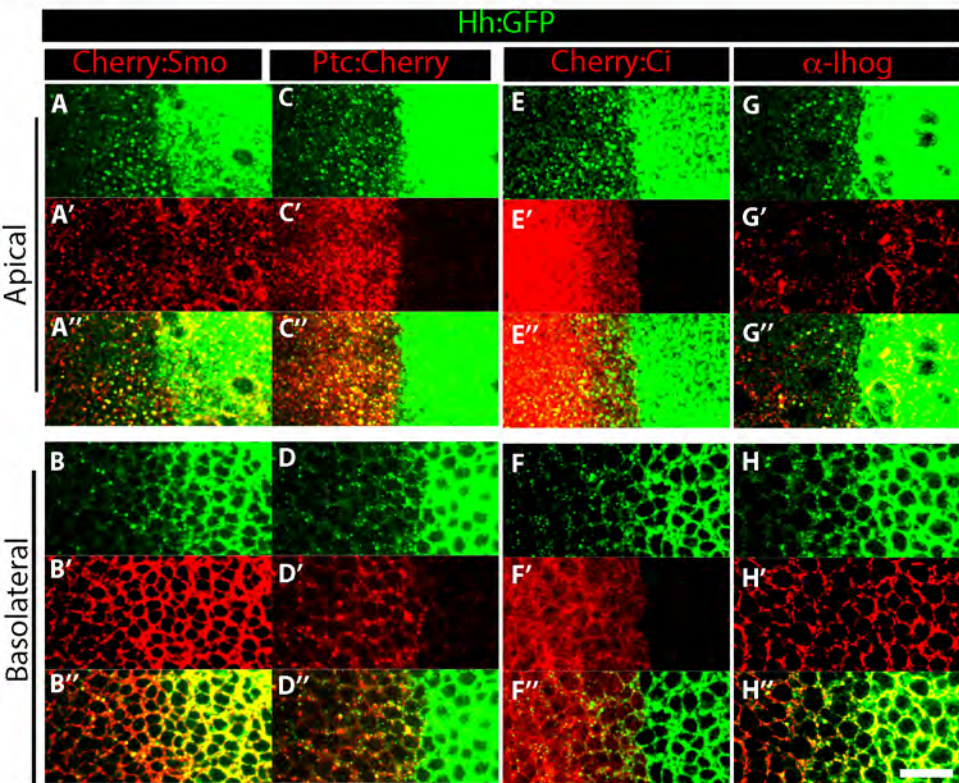

Supplement: 1 [file NIHPP2026.01.08.698469v1-supplement-1.pdf]
